# Obestatin: canonical and unexpected functions

**DOI:** 10.1007/s11154-026-10021-0

**Published:** 2026-03-05

**Authors:** Icia Santos-Zas, Uxia Gurriaran-Rodriguez, Tania Cid-Diaz, Saul Leal-Lopez, Felipe F. Casanueva, Yolanda Pazos-Randulfe, Jesus P. Camiña

**Affiliations:** 1 Group of Myology, Health Research Institute of Santiago de Compostela (IDIS), Santiago de Compostela, Spain; 2 CIC bioGUNE, Bizkaia Technology Park, Derio, 48160 Spain; 3 Department of Pathology and Laboratory Medicine and Sandra and Edward Meyer Cancer Center, Weill Cornell Medicine, New York, NY 10065 USA; 4 Group of Translational Research in Digestive System Diseases, IDIS, Santiago de Compostela, Spain; 5 Epigenomics in Endocrinology and Nutrition Group, Epigenomics Unit, IDIS, Complejo Hospitalario Universitario de Santiago de Compostela, University of Santiago de Compostela, Santiago de Compostela; CIBER de Fisiopatologia de la Obesidad y Nutricion (CIBERobn), Instituto de Salud Carlos III,, Madrid , Spain

**Keywords:** Obestatin, GPR39, Skeletal muscle, Atrophy, Wasting muscle

## Abstract

The functions of appetite-regulating hormones have been studied for decades with the aim of finding a solution to the problem of obesity. Among these molecules, a small peptide called obestatin has emerged as an anorexigenic hormone, with an antagonistic effect to the hunger hormone ghrelin. After years of controversy regarding its function in food intake and the establishment of its receptor, GPR39, obestatin is currently being proposed as a powerful therapeutic candidate for pathologies associated with skeletal muscle. Several studies have demonstrated its key role as a regulatory peptide in myogenesis, thereby increasing regeneration in acute muscle damage. Obestatin promotes vascularization and reduces fibrosis in regenerated tissue, while also increasing muscle strength in muscle atrophy pathologies associated with glucocorticoid treatment and Duchenne muscular dystrophy. This review describes the main mechanisms and signaling pathways regulated by the obestatin peptide in muscle pathology.

## Obestatin, an appetite-regulating hormone

The study of obesity, the silent pandemic of the 21 st century, has led to the discovery of what are known as “appetite-regulating hormones.” These hormones are a group of molecules secreted by the intestinal tract and the adipose tissue. Their main function is to transmit all the information about the body’s energy status to the brain, thereby maintaining the balance between food intake and caloric expenditure [1]. Chronic dysregulation of this communication system is a major feature in the pathophysiology of obesity, resulting in imbalances in appetite and/or satiety. Among these chemical messengers are orexigenic molecules that stimulate appetite, like ghrelin, and anorexigenic molecules which promote satiety, like leptin, glucagon-like peptide-1 (GLP-1) or insulin [2–4]. These molecules have enabled the development of therapies aimed at controlling food intake, mainly through the use of satiety hormone analogues.

In 2005, the discovery of a new appetite-regulating hormone called obestatin was published in the prestigious journal Science [5]. At that time, there was considerable confusion and mystery surrounding the function of ghrelin peptide, a well-known hunger hormone. The complete deletion of the ghrelin gene, *GHRL*, did not produce a significant loss of appetite in animals nor did it have an impact on body weight, which was striking given ghrelin’s established role as a potent appetite stimulant to date [6]. At this confusing juncture, Zhang and colleagues conducted a study of the ghrelin gene and its precursor peptide, preproghrelin [5]. Using a bioinformatic approach, they analyzed the preproghrelin messenger RNA (mRNA) demonstrating that the *GHRL* gene encoded not only ghrelin, but also a 23-amino acid “sibling peptide” that they named obestatin. Following posttranscriptional and posttranslational processing (alternative splicing, RNA processing, and modifications of prepropeptide residues), preproghrelin precursor gives rise to both hormone peptides: ghrelin and obestatin. In an effort to determine the functions of this new peptide, Zhang and colleagues injected obestatin into rats. They observed a decrease in their food intake, a delay in gastric emptying and lower jejunal motility [5, 7, 8]. Obestatin was therefore postulated as a new appetite-regulating hormone with an antagonistic function to the orexigenic action of ghrelin, initiating the substantial controversy that has surrounded this small peptide for decades.

## Obestatin/GPR39 system: a source of conflict

**Fig. 1:**
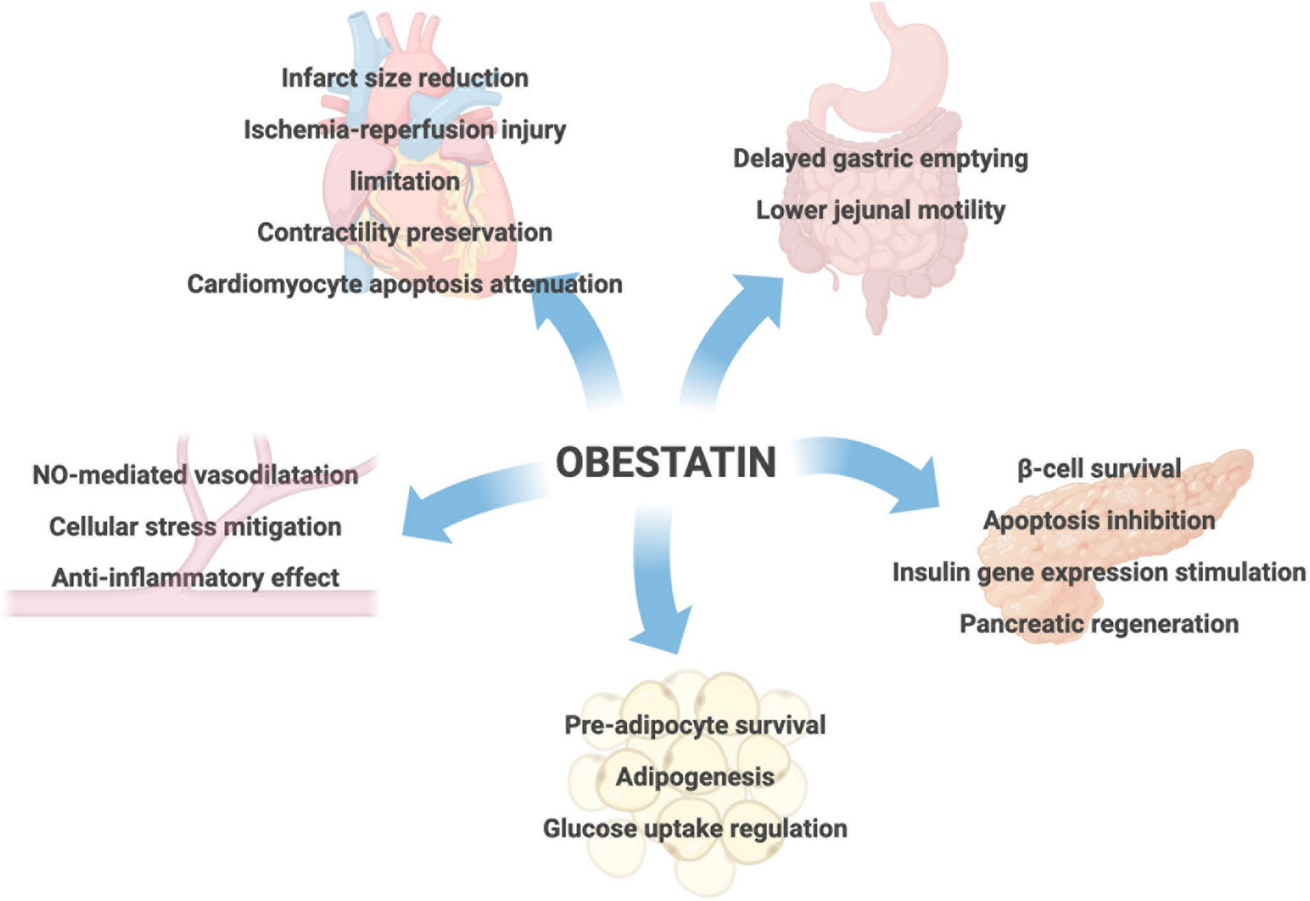
Canonical functions and tissue-specific actions of obestatin. Obestatin acts on several peripheral tissues-such as the gastrointestinal tract, pancreas, white adipose tissue, heart, and vasculature-modulating key functions related to metabolism, energy balance, and tissue protection. Figure created using BioRender

Following its initial discovery in 2005, it was determined that obestatin expression in humans was mainly located in the gastrointestinal tract. To a lesser extent, this small hormonal peptide has also been found in the spleen, pancreas, prostate, testis, thyroid and skeletal muscle [9–11]. Structurally, obestatin adopts an α-helical conformation that enhances its stability and biological activity [12]. Shortly after its identification, obestatin became a very controversial peptide. Although it was postulated originally as an anorexigenic molecule with antagonistic functions to ghrelin, a number of studies failed to reproduce any effect on energy or homeostasis balance questioning its action as the promising opponent of the hunger hormone [13–15]. Instead, several pieces of evidence suggest a more complex, context-dependent relationship between obestatin and ghrelin, characterized by both synergistic and complementary actions as well as opposing functions [16–28]. Despite not having a clear opposite effect to ghrelin, some studies conducted in humans show that circulating obestatin levels are generally reduced in obesity, type 2 diabetes, insulin resistance, and metabolic syndrome, correlating inversely with body mass index (BMI), glucose, insulin, leptin, and glycated hemoglobin (HbA1c) [29–35]. Obestatin also exerts an effect on adipose tissue by promoting pre-adipocyte survival, supporting adipogenesis, and regulating glucose uptake via GLUT4 translocation [36–39]. In addition, it plays an important role in glucose and lipid metabolism. These metabolic actions are cell-type and context-dependent. Beyond its role in feeding regulation and its direct association with obesity and adipose tissue, obestatin participates in a wide range of physiological processes and becomes dysregulated under pathological conditions [40]. Indeed, it enhances β-cell survival, inhibits apoptosis, stimulates insulin gene expression, and supports pancreatic regeneration. In diverse experimental models of diabetes, obestatin preserves islet mass, improves glucose tolerance, and augments insulin secretion [41]. On the other hand, obestatin has also been implicated in cardiovascular physiology. Although its impact on blood pressure regulation remains inconclusive, it consistently exerts endothelial-protective effects by promoting nitric oxide–mediated vasodilation [42, 43], mitigating hyperglycemia-induced cellular stress, and suppressing pro-inflammatory activation [44]. At the cardiac level, obestatin reduces infarct size, limits ischemia–reperfusion injury, preserves contractility, and attenuates cardiomyocyte apoptosis [45, 46]. Elevated obestatin levels have been reported in conditions such as chronic heart failure–related cachexia and cardiorenal syndrome, possibly reflecting compensatory mechanisms [47, 48]. An overview of the canonical functions and tissue-specific actions of obestatin is provided in Fig. 1.

The second controversial aspect related to the obestatin peptide has been the establishment of its receptor. In 1997, two new G protein-coupled receptors, GPR38 and GPR39, were isolated and cloned together from human genomic DNA libraries as orphan GPCRs. GPR38 expression was restricted to the stomach, thyroid, and bone marrow only, whereas GPR39 transcripts were detected in many different tissues such as hypothalamus, white adipose tissue, intestine, stomach, pancreas, thyroid, colon and recently in mouse and human skeletal muscle [49, 50]. Two years later, GPR38 was recognized as the motilin receptor [51]. Conversely, GPR39 was considered an orphan GPCR until 2005, when Zhang and colleagues discovered obestatin. Based on data from radiolabeled ligand binding assays, Zhang´s group proposed obestatin as the ligand for GPR39 [5]. Immediately, conflicting opinions appeared in this regard, questioning this ligand-receptor relationship, especially after Zhang’s group failed to reproduce the original results of their research [52]. These events supported the need to continue the search for the GPR39 ligand. In this scenario, the idea of GPR39 as a Zn^2+^-sensing receptor began to gain strength despite conflicting results regarding Zn^2+^ physiological function in terms of cell proliferation and survival [53–56]. This proposal was challenged when two different studies postulated Zn^2+^ as a secondary player controlling GPR39 signaling. Specifically, these works demonstrated that obestatin signaling through GPR39 required in some cases epidermal growth factor receptor (EGFR) transactivation via Zn^2+^-dependent matrix metalloproteinases (MMPs) activity [22, 57]. In this uncertain environment, the search for the obestatin receptor was also the focus of many studies, with glucagon-like peptide-1 receptor (GLP1R) being the main candidate in line with obestatin-positive effects on glucose and lipid metabolism [41]. Over time, this proposal was strongly questioned due to the inability of obestatin to bind to INS-1 beta-cells or HEK cells overexpressing GLP-1R or to displace GLP-1 binding to these cells [15]. In 2008, Zhang’s group once again took the initiative in the field proving that previous inconsistent binding of obestatin to GPR39 was due to the loss of obestatin bioactivity after its poly-iodination. Mono-iodinated obestatin bound to HEK293 cells after being transfected with GPR39 plasmids [8]. In addition, studies in bone metabolism and in pregnancy have shown a correlation in expression levels of both GPR39 and obestatin [58, 59]. An elegant structural study using nuclear magnetic resonance imaging analysis has shown not only that obestatin presents a necessary domain for GPR39 activation, but also that the biological effect of the obestatin peptide is species-dependent [60]. The fact that human and mouse obestatin show differences in their activity could explain many inconsistent results described at that time. Finally, using different GPR39-knocked down cell lines by siRNA (3T3-L1 preadipocytes, C2C12 mouse myoblasts, human gastric adenocarcinoma cells, AGS, or human immortalized myoblasts) it has been demonstrated that the impairment of obestatin signaling occurs in the absence of the GPR39 receptor [24, 56, 60, 61]. In 2015, co-immunoprecipitation assays using hemagglutinin (HA)-tagged obestatin in C2C12 myoblasts conclusively determined that obestatin co-immunoprecipitated specifically with GPR39, showing the binding of GPR39-obestatin and proving definitively that obestatin is the cognate ligand of the GPR39 receptor in skeletal muscle cells [61].

## Obestatin: a peptide that regulates skeletal muscle

### Skeletal muscle: structure and function

Skeletal muscle is the most abundant human tissue, comprising approximately 40% of total body weight. It is a well-organized tissue made up of myofibers, which are the result of the fusion of myogenic precursor cells. One of the most relevant characteristics of this tissue is the capability of generating forces through its contractility. This property makes it an indispensable tissue for maintaining an upright posture, producing movement and respiration. However, skeletal muscle is much more than just a structural organ, as it develops important roles in many other physiological activities, such as metabolism, thermogenesis, and secretion of different peptides [62–64]. From the perspective of obesity research, there is clear evidence of the important role that fat-free compartments play in appetite control. Numerous studies have shown the positive relationship between lean body mass, energy intake and hunger [65, 66]. The simplistic model of appetite control, which has only taken into account the relationship between adipose tissue and the brain, has given way to a global and multifactorial model, dependent on the maintenance and activity of muscle mass as a regulator of energy intake and appetite. Thus, maintenance of skeletal muscle health is of vital importance. Accordingly, the study of pathologies related to the structure and/or functioning of the skeletal muscle, i.e., myopathies, acquires a relevant interest. These disorders can be triggered by inherited genetic diseases (such as dystrophies or mitochondrial myopathies), metabolic errors (such as carnitine deficiency), certain drugs and toxins, infections, inflammation, or even hormonal irregularities [67]. Currently, two muscle disorders have acquired significant importance due to their high prevalence in society and to their impact on the quality of life and life expectancy of patients: sarcopenia and cachexia, both muscle wasting conditions related to aging and to underlying illnesses such as cancer, acquired immune deficiency syndrome (AIDS) or congestive heart failure (CHF) among others [68–71]. All these myopathies and skeletal muscle disorders are characterized by their primary symptom: muscle weakness due to dysfunction of the muscle fiber and severe wasting of skeletal muscle. Notably, the current clinical approaches for many of these pathologies are still limited. In this context, the need to identify and develop new therapeutic strategies for the treatment of disorders associated with skeletal muscle is clear. These therapeutic approaches should mitigate or counteract muscle weakness and wasting considering the characteristics of each of the pathologies. Molecular and cellular knowledge of the degenerative and inflammatory processes that cause damage to muscle fibers, as well as remodeling processes, is essential for finding new therapeutic targets. Nowadays, several autocrine/paracrine signals have been described as important players in the process of muscle growth and in the control of myogenesis. Different hormones and growth factors have been described as important mediators of skeletal muscle adaptations, such as myofiber regeneration or the plasticity of the muscle fibers [72, 73]. Given the strong link between fat mass and muscle and their complex interdependence, studying the role of appetite-regulating hormones on muscle mass could lead to significant advances in pathologies associated with skeletal muscle wasting.

### Skeletal muscle regeneration

Skeletal muscle is a postmitotic tissue with an extraordinary regenerative and repair capacity after severe traumas or different kinds of myopathies. This repair process is a highly orchestrated mechanism, which concludes with the formation of a functional contractile system. The main phases of skeletal muscle repair are: (1) the degenerative phase which begins with rupture and fiber necrosis, activating calcium-dependent proteolysis after the disruption of the myofiber sarcolemma; (2) the inflammatory response induced by the increased proteolysis and cell death as a consequence of necrotic changes. This response entails the release of chemotactic inducers and the infiltration of different types of inflammatory cells into the injured tissue. Neutrophils and macrophages are the main players, removing fragments of damaged tissue and releasing cytokines, growth factors and enzymes involved in the repair process [74, 75]; (3) the regeneration phase is mediated by specific resident muscle stem cells (MuSCs) called satellite cells which are unipotent muscle precursor cells. These MuSCs are located in a specialized niche, embedded between the basal lamina of the muscle and the sarcolemma of the myofiber. Under physiological conditions MuSCs remain in a non-mitotic or quiescent state, but after injury this group of cells is rapidly activated and driven out of their quiescent state [76, 77]. Once MuSCs are activated, they start to proliferate giving rise to myoblasts that migrate to the damaged area and differentiate into myotubes. These new myotubes can fuse with each other or with existing myofibers to repair the injured muscle. The correct progression of the myogenic program is controlled by several myogenic regulatory factors (MRFs), including myogenic factor 5 (Myf5), myoblast determination protein 1 (MyoD), myogenin and the myogenic regulatory factor MRF4 [76, 78]. Although all MuSCs are characterized by the expression of the canonical regulator Pax7 [79], these cells are a heterogeneous population, with different expression profiles, variations in their proliferation and differentiation potentials and with differences in their stemness [80, 81]. Interestingly, MuSCs can both replicate themselves (self-renew) and/or give rise to functional progeny (differentiate). The self-renewal ability is essential for proper muscle homeostasis, as it allows the maintenance of the muscle progenitor cell pool [82]. MuSCs regulate this ability by performing two different types of divisions: symmetric divisions will result in two identical daughter stem cells that replenish their pool. However, asymmetric divisions generate one stem cell and another committed progenitor that participates in muscle regeneration [76, 78]. (4) The last phase of muscle repair, remodeling, consists of the reestablishment and maturation of skeletal muscle architecture, increasing the size of newly formed myofibers, establishing neuromuscular junctions and promoting revascularization.

### Obestatin/GPR39 system and skeletal muscle regeneration

Given the complexity of the skeletal muscle regenerative system, different strategies have been proposed over the years to address the regenerative problem without obtaining clear benefits in many of the disorders. From a pharmacological point of view, an ideal drug would be that one that has the capacity to act favorably on several of the muscle-repair-stages described previously. Specifically, modulation of MuSC activity through a factor that allows enhancement of their regenerative potential without exhausting the MuSC pool would be one of the main characteristics of any pharmacological agent. Based on this, it is worth noting the potential of the obestatin/GPR39 system as a regulator of the myogenic process. In 2012, the first results were published describing the implication of this system in skeletal muscle regeneration [18]. Adult healthy skeletal muscle expresses obestatin and GPR39. Interestingly, using rat and mouse models it was revealed that the obestatin/GPR39 system was overexpressed after an acute muscle injury. Furthermore, in vitro experiments have shown that obestatin was rapidly upregulated during myogenesis. This peptide was especially expressed in mouse, rat and human myotubes, whereas GPR39 was expressed equally in both myoblasts and myotubes [18, 57, 61]. In addition, blocking obestatin expression through preproghrelin siRNA or blocking obestatin action using antibodies during myogenesis resulted in a decrease in the expression levels of myogenic markers, such as myogenin and myosin heavy chain (MHC). Similar effects were obtained by silencing GPR39, which demonstrated the autocrine role of this system in the myogenic process. Furthermore, exogenous administration of obestatin promotes myogenic differentiation and the fusion of myoblasts. These results were further supported by different in vivo rat and mouse models with acute skeletal muscle damage: obestatin overexpression in skeletal muscle, obestatin intramuscular injection or obestatin subcutaneous infusion improved skeletal muscle regeneration [18, 82–84]. The obestatin/GPR39 system regulates different stages of myogenesis by the upregulation of markers related to MuSC activation, Pax7 and Myf5, and markers associated with myogenesis, such as MyoD, myogenin, Myf6 and MHC. The increased expression of the marker Pax7 reflects a clear obestatin effect on MuSC activation, and accordingly, on myogenic specification and self-renewal of these cells. In addition to the control exerted on myogenic regulatory factors, obestatin also increases the mitogenic activity of differentiating myoblasts, their migration, and their fusion, fostering a higher hypertrophic growth of myotubes [18, 57]. Furthermore, the obestatin action is not restricted to the regenerative stage, but also promotes the microvascularization of regenerated tissue and reduces the interstitial fibrosis, regulating the microenvironmental remodeling [61].

#### Obestatin signaling in human myogenesis

Using a stable immortalized human myoblast cell line and different cellular strategies it has been possible to establish the mechanism by which obestatin regulates human myogenesis [57]. Both, G-protein dependent and β-arrestin-dependent mechanisms have been described as main players controlling obestatin-related myogenic action. Early obestatin action during myogenic differentiation is determined by G-protein-dependent activation, defining the intricate pathway related to mitogen-activated protein kinases (MAPKs): extracellular signal‑regulated protein kinase 1/2 (ERK1/2), c-Jun N-terminal kinases (JNK1-3), and p38 mitogen-activated protein kinase. The ERK1/2 pathway is essential for cell proliferation and accordingly, obestatin stimulates ERK1/2 activity during the first stages of myogenesis, consistent with a higher proliferative state just prior to undergoing the transition to differentiation. After the first proliferative stage of the myogenic process and along with a decrease in ERK1/2 bioactivity, obestatin regulates transactivation of EGFR through the β-arrestin signal complex. A signalplex composed of β-arrestin 1 and 2, GPR39, and proto-oncogene tyrosine-protein kinase (Src) allows the initiation of EGFR transactivation through metalloproteinases, promoting: (1) JNK/c-Jun, p21 and p57 signaling and, consequently, cell cycle exit; and, (2) Akt, p38, and CAMKII signaling, controlling recruitment and fusion of myoblasts aimed at the formation of multinucleate myotubes [57].

### Obestatin, muscle fiber plasticity and muscle functionality

**Fig. 2:**
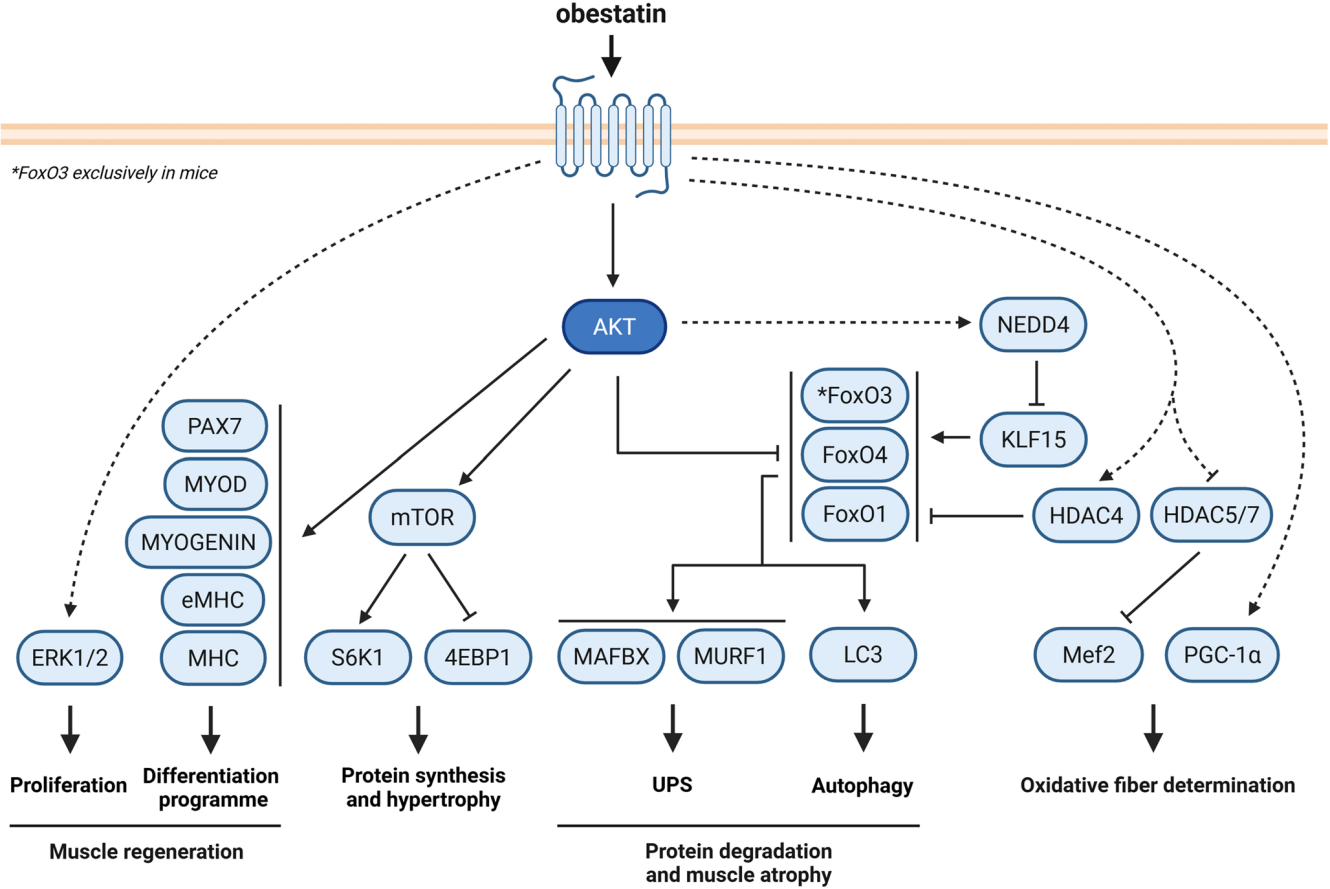
Diagram shows the main signaling pathways regulated by obestatin in skeletal muscle. The ERK1/2 signaling node regulates myoblast proliferation. The Akt pathway regulates the complex differentiation program and the protein synthesis. Muscle fiber-type determination is delineated by Mef2 and PGC-1α. Additionally, obestatin inhibits FoxO activity in multiple ways. It promotes the post-translational modification of FoxO proteins, including their phosphorylation by Akt, and modifies FoxO protein levels via NEDD4-dependent ubiquitination of KLF15. Figure created using BioRender

Skeletal muscle groups are made up of bundles of muscle fibers assigned to different identity classifications or types, with characteristic movement rates, responses to neural inputs, and metabolic styles [85]. Muscle fibers can be classified using different criteria: degree of fatigability during sustained activity (fatigable vs. fatigue-resistant), predominance of certain metabolic pathways (oxidative vs. glycolytic), calcium handling (fast vs. slow) [86] and protein isoform expression [87]. Based on specific MHC isoform content and expression, myofibers are classified into type I and type II. Type II fibers can be further differentiated into type IIa, IIb, and IId/x [88]. Type I fibers (slow-twitch, oxidative) present high resistance to fatigue by being rich in greater mitochondria. They metabolize lipids as a source of energy. These fibers are required for the maintenance of posture and tasks involving endurance. Conversely, type IIb fibers (fast-twitch, glycolytic) use glycogen and glucose as fuel, and they are required for movements involving strength and speed. Type IIa (fast-twitch, oxidative) and type IId/x (fast-twitch, oxido-glycolytic) present intermediate contractile speeds compared to type I and IIb fibers [89]. All these different types of fibers can change their phenotype modifying their contractile and metabolic properties [90], and converting from one type to another in response to several environmental and physiological demands [88, 91, 92]. Several lines of evidence indicate that changes in intracellular calcium concentration activate signaling pathways that trigger modifications in gene expression and control fiber remodeling. In this context, it has been shown that obestatin controls fiber type determination, prioritizing the oxidative over the glycolytic phenotype [84]. Using a mouse model of skeletal muscle regeneration after acute trauma, it has been revealed that obestatin significantly increases muscle strength in a dose-dependent manner. The improvement in mean twitch and tetanic forces is further accompanied by an increase in the cross-sectional area of the myofibers and in the wet weight of tibialis anterior (TA) under obestatin stimuli. This increase in force is due to a shift in fiber type toward an oxidative phenotype, with an increase in the number of oxidative fibers. Furthermore, obestatin increases the number of type I fibers and reduces the appearance of type IIa and IIx. The molecular mechanisms used by obestatin to exert this function on fiber plasticity are based on the upregulation of myocyte enhancer factor-2 (MEF2) factor as a result of the nuclear export of histone deacetylase II (HDACII) through the upregulation of calcium-regulated protein kinases, protein kinase D (PKD) and calcium calmodulin-dependent protein kinase II (CaMKII). This well-orchestrated process allows the sustained activation of MEF2-dependent genes, such as slow-twitch contractile protein genes, myoglobin, and slow troponin [93, 94]. Obestatin signaling regulates the expression of different regulators of mitochondrial function, such as cytochrome C, uncoupling protein 1 (UCP-1) and carnitine palmitoyltransferase 1 (CPT-1), and the peroxisome proliferator-activated receptor gamma coactivator-1 alpha (PGC-1α), associated with mitochondrial biogenesis, which reflects enhanced mitochondrial capacity and oxidative metabolism. Similarly to what is described in mice, obestatin favors a shift toward an oxidative phenotype, without significant changes in MEF2 expression or CaMK activation [84]. Figure 2 shows an overview of the intracellular signaling pathways regulated by obestatin in skeletal muscle.

### Obestatin/GPR39 system and glucocorticoid-induced muscle atrophy

Muscle atrophy is the wasting or thinning of muscle mass [95, 96]. This phenomenon occurs when protein degradation exceeds protein synthesis. Increased oxidative stress, inflammation, and decreased mitochondrial function are considered important upstream signals of muscle atrophy caused by several pathologies, such as chronic obstructive pulmonary disease, cachexia, diabetes, renal failure and sepsis, as well as a consequence of direct inactivity, immobilization or denervation simply [97]. The imbalance between protein synthesis and protein degradation implies a complete deregulation of the main systems that control the catabolic and anabolic activities: the ubiquitin proteasome system (UPS) [98], the autophagy lysosome system (ALS) [98, 99], the caspase system [100], the calpain system [101] and the mammalian target of rapamycin (mTOR) signaling pathways [102, 103]. Several pathological states characterized by muscle atrophy are associated with an increase in circulating glucocorticoids and poor patient prognosis, making it an important target for treatment. The activation of the obestatin/GPR39 system protects against glucocorticoid-induced atrophy by regulating Akt, PKD/PKCµ, CAMKII and AMP-activated protein kinase (AMPK) signaling and its downstream targets in controlling protein synthesis, UPS, and ALS in mouse cells [104]. Obestatin signaling negatively modulates GR-mediated gene expression, and this action derives from the regulation of forkhead box O (FoxO) transcription factors at both translational and post-transcriptional levels. At the post-translational level, FoxO family is negatively regulated by the Akt pathway in response to obestatin. Phosphorylation of FoxO factors by Akt causes the sequestration of FoxO in the cytoplasm, thereby preventing FoxO factors from transactivating their target genes in the nucleus. Neither FoxO1 nor FoxO3 is involved in the response to obestatin via phosphorylation by Akt activation. In contrast, FoxO4 is the main obestatin-responsive isoform, thus supporting an isoform-specific mode of regulation. At the transcriptional level, the effect of obestatin on FoxO factors is mediated via krüppel-like transcription factor 15 (KLF15) ubiquitination by the E3 ubiquitin ligase neural precursor cell expressed developmentally downregulated protein 4 (NEDD4). Interestingly, the NEDD4 expression under obestatin signaling correlates with a regression of the atrophic process, concomitant with a decreased expression of KLF15 protein. Thus, NEDD4 controls FoxO and the expression of atrophy-related genes, i.e. atrogenes [83]. Both modes of FoxO regulation by obestatin signaling, together with the enhancement of the protein synthesis pathway, restore basal homeostasis conditions. These actions highlight the potential therapeutic relevance of the obestatin/GPR39 system for the fine-tuning of muscle mass in conditions characterized by muscle atrophy associated with increased circulating glucocorticoid levels.

### Obestatin and Duchenne muscle dystrophy

In 1968 L. Kunkel described the *DMD* gene, the longest in the human genome. The product of this gene, dystrophin [105], is a cytoskeletal protein with a crucial role in skeletal and cardiac muscles. Dystrophin is associated with a large macromolecular complex of proteins (dystroglycans, sarcoglycans, dystrobrevins, syntrophins and sarcospan) forming what is known as the dystrophin-glycoprotein complex (DGC). The main function of the DGC is to link cytoskeletal actin to the extracellular matrix. However, the function of this complex is not solely based on the maintenance of the structural integrity of the muscle. Proteins of the DGC are involved in mechanotransduction and different signaling pathways of vital importance for the correct biological functioning of muscle cells [106]. Loss of dystrophin produces sarcolemma instability and myofiber damage as muscle fibers detach from the laminin-rich basal lamina during contraction [107]. In addition, the absence of dystrophin leads to chronic inflammation, progressive fibrosis, and dysfunction of MuSC. In this regard, mutations in *DMD* gene, which encodes the central protein of the DGC, dystrophin, cause Duchenne muscular dystrophy (DMD). DMD, an X-linked disorder, is a severe, progressive, muscle-wasting disease, affecting 1 in 5,000 live male births [108, 109]. The severity of DMD depends on the mutation type [110]. Affected boys develop the earliest symptoms, such as frequent falls due to muscle weakness, around 2–3 years of age. Most patients lose the ability to walk around 10–12 years of age, becoming wheelchair dependent and succumbing to respiratory and cardiac failure between 20 and 40 years of age with optimal care [111, 112]. Nowadays, there is still no cure for DMD. At present, glucocorticoids remain the only treatment unequivocally shown to slow disease progression, despite the adverse effects associated with their long-term use [113, 114]. The development of gene therapies [115], cell-based therapies [116], and other therapeutic approaches [116, 117] are still promising strategies that need to evolve before they become viable proposals [118]. In this context, obestatin arises as therapeutic approach aimed at enhancing endogenous satellite cell function and protecting the fibers from degenerating [84]. Obestatin improves muscle strength ameliorating the DMD phenotype in the dystrophin deficient *mdx* mouse. In addition to the established role in the control of muscle regeneration, obestatin remodels skeletal muscle toward an oxidative phenotype, with improved muscle strength and reduced skeletal muscle pathology. This action is mediated by both HDAC/MEF2 and PGC1α mechanisms, thereby controlling the establishment of oxidative muscle fibers. Furthermore, obestatin signaling regulates muscle atrophy through regulation of ubiquitin E3-ligase expression, muscle atrophy F-box (MAFbx), and muscle RING-finger protein-1 (MuRF1), through inactivation of FoxO4 and FoxO1. Notably, obestatin signaling stabilizes the sarcolemma of *mdx* skeletal muscle through the upregulation of utrophin, α-syntrophin, β-dystroglycan, and α7β1-integrin proteins [84]. This action stabilizes the link between the sarcolemma and the extracellular matrix providing protection to the myofibers from contraction-induced muscle injury. This observation correlates with the decrease in the levels of serum creatine kinase (CK), alanine aminotransferase (ALT), and aspartate aminotransferase (AST), which are signs that indicate a partial rescue of muscle tissue necrosis [119, 120]. Furthermore, obestatin reduces muscle fibrosis. Besides the *mdx* model, obestatin favors the myogenic program in human DMD through the recruitment and fusion of DMD myoblasts into myotubes. Furthermore, obestatin acts to increase levels of both utrophin and α7β1 integrin complexes during the myogenic differentiation. These data position obestatin as a potential DMD therapeutic candidate not only to slow the muscle damage but also as part of combinatorial treatment strategies in the dystrophic muscles [84].

### Obestatin and cell therapy related to myopathies

**Fig. 3:**
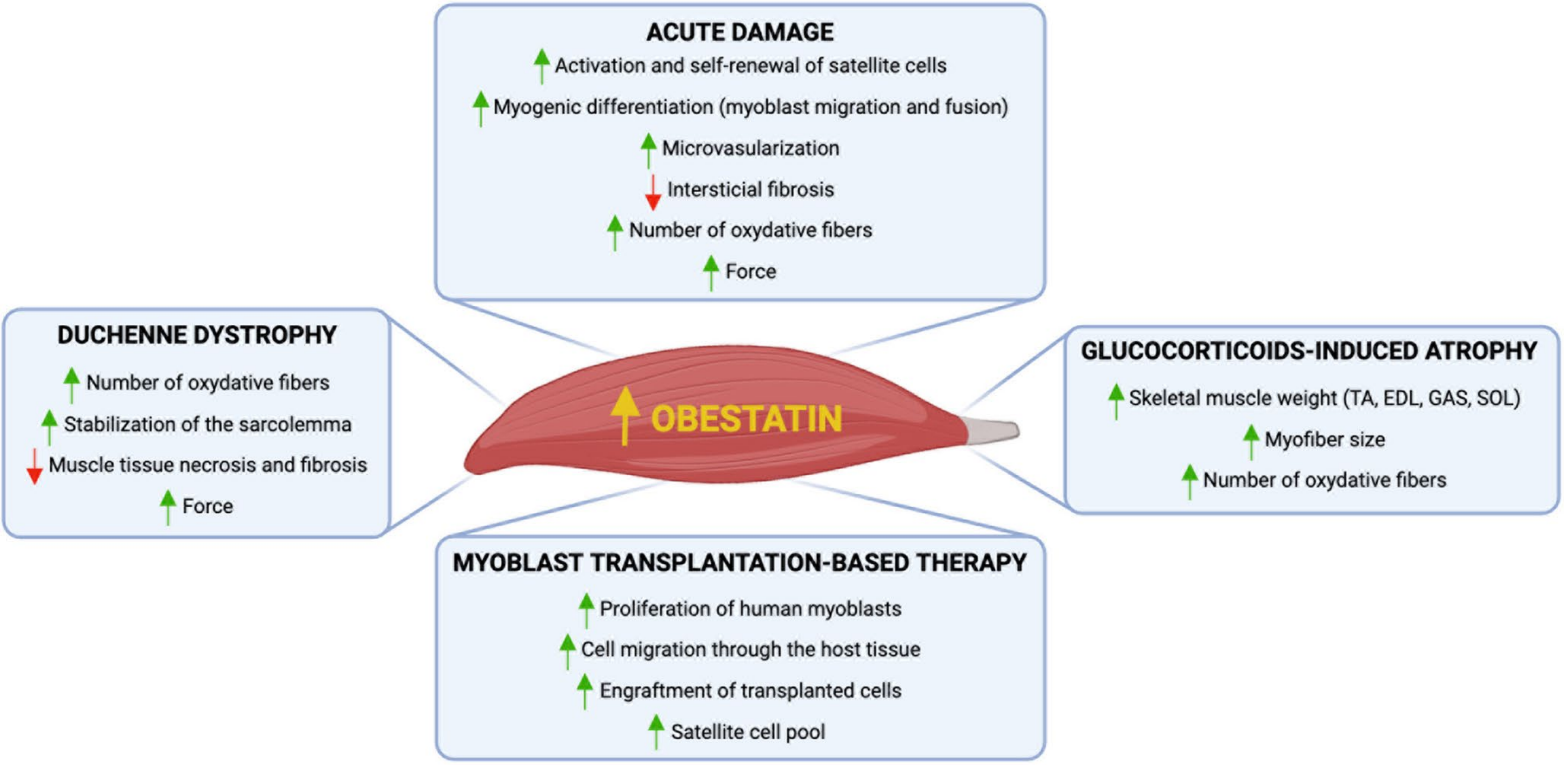
Schematic representation of the effects of obestatin on skeletal muscle under different conditions. Figure created using BioRender

The vast regenerative capacity of the skeletal muscle, together with the existence of muscle stem cells, makes muscle tissue an ideal target for cell therapy. The transplantation of various cell candidates of myogenic or non-myogenic origin, with the aim of repairing muscle tissue has been a widely investigated therapeutic strategy. Although promising, this type of therapy has not been fruitful in clinical practice. The low therapeutic efficacy results from biological and technical limitations, such as the poor survival of transplanted cells, their lack of dispersion from the injection site and the immunological rejection by the host [121, 122]. The use of a pharmacological approach that targets these drawbacks together with the cell therapy itself, can improve the efficacy of this type of intervention. Thus, combined therapies are postulated as promising therapeutic interventions to address muscle disorders. The data presented so far, position the obestatin/GPR39 system as a potential therapeutic target, not only as an ameliorative treatment to slow the muscle damage but also as part of combinatorial treatment strategies [123]. Thus, obestatin-mediated fiber restoration could help to mitigate the loss of therapeutic cell content in cell therapy and guarantee a higher therapeutic benefit in the restoration of muscles in patients. Furthermore, any intervention upon the donor cells focused on enhancing in vivo survival, proliferation, and expansion could improve the effectiveness of such therapies in regenerative medicine. Indeed, activation of obestatin signaling proves to improve the outcome of myoblast-based therapy by xenotransplanting primary human myoblasts into immunodeficient mice [123]. Obestatin stimulates the proliferation of transplanted human myoblasts by delaying myogenic differentiation and, thus, increasing the number of cells capable of colonizing a much larger area within the recipient’s muscle. Obestatin treatment results in increased cell migration through activation of Dishevelled Segment Polarity Protein 2 (Dvl2) and the small GTPase Rho signaling pathway. Obestatin-stimulated myoblasts maintain their ability to differentiate; moreover, obestatin treatment leads to a hypertrophic response in the human-derived regenerating myofibers through the activation of the protein synthesis pathway. Notably, the capacity to reconstitute the satellite cell pool from transplanted cells was enhanced by obestatin signaling increasing the number of human Pax7 + cells in the satellite cell compartment. Therefore, obestatin not only allows reconstitution of mature muscle fibers, but also replenishes the pool of muscle precursors by engrafted donor cells, thus seeding a reserve pool of cells that may be recruited for subsequent rounds of muscle repair. This action advocates its potential for long-term treatment of muscle diseases. Combined, the activation of the obestatin/GPR39 pathway results in an overall improvement in the efficacy of reconstituting the host skeletal muscle. Figure 3 provides a schematic overview summarizing the effects of obestatin on skeletal muscle.

### Concluding remarks

The pathologies associated with skeletal muscle have emerged in recent decades as a problem with a high impact on the health and quality of life of affected patients. In addition, the high incidence of myopathies such as cachexia or sarcopenia, represents an enormous cost for health systems today. Therefore, it is necessary to search for therapeutic targets that allow the structural integrity of the muscle to be reestablished through the induction of the regenerative process of the muscle tissue itself, which is essential when establishing new therapeutic approaches. The obestatin-related regenerative properties not only alleviate the symptoms associated with the weakening or loss of muscle mass described in these types of disorders but also have a direct effect on the recovery of muscle strength. In this context, obestatin administration, alone and/or in combination with other therapies, represents a clear translational potential for humans, offering unique opportunities for muscle regenerative medicine.

## Data Availability

No datasets were generated or analysed during the current study.

## Abbreviations and acronyms

AIDS: acquired immune deficiency syndrome
ALS: autophagy lysosome system
ALT: alanine aminotransferase
AMPK: AMP-activated protein kinase; AST: aspartate aminotransferase
BMI: body mass index
CaMKII: calcium calmodulin-dependent protein kinase II
CHF: congestive heart failure
CPT1: Carnitine palmitoyltransferase 1
DGC: dystrophin glycoprotein complexes
DMD: Duchenne muscular dystrophy
DVL2: Dishevelled Segment Polarity Protein 2
FoxO: forkhead box O
GHRL: Ghrelin
GLP-1: glucagon-like peptide-1
GPCRs: G protein-coupled receptors
HDAC: Histone deacetylase
KLF15: Krüppel-like factor 15
MAFbx: muscle atrophy F-box
MEF2: Myocyte Enhancer Factor-2
MHC: myosin heavy chain
MuRF1: muscle RING-finger protein-1
MMPs: Matrix metalloproteinases
MRFs: myogenic regulatory factors
mTOR: mammalian target of rapamycin
MuSCs: muscle stem cells
Myf5: myogenic factor 5
MyoD1: myoblast determination protein 1
NEDD4: neural precursor cell expressed developmentally downregulated protein 4
PGC-1α: peroxisome proliferator-activated receptor gamma coactivator-1 alpha
PKD: protein kinase D
SCK: serum creatine kinase
TA: tibialis anterior
UCP1: Uncoupling protein 1
UPS: ubiquitin proteasome system
